# Complete genome sequence of Australian soil bacterium *Rouxiella badensis* DAR84756 resolved with Oxford Nanopore long-read and Illumina sequences

**DOI:** 10.1128/mra.00857-23

**Published:** 2023-12-01

**Authors:** Saina Paul, Peter J. Anderson, Gregg J. Maynard, Michael Dyall-Smith, Timothy Kudinha

**Affiliations:** 1 School of Dentistry and Medical Sciences, Charles Sturt University, Leeds Parade, Orange, New South Wales, Australia; 2 Veterinary Biosciences, Melbourne Veterinary School, Faculty of Science, University of Melbourne, Parkville, Victoria, Australia; 3 NSW Health Pathology West, Orange, New South Wales, Australia; University of Southern California, Los Angeles, California, USA

**Keywords:** soil, rhizosphere, Gram negative

## Abstract

The complete genome sequence of the bacterium *Rouxiella badensis* DAR84756, isolated from soil in Orange, NSW, Australia, was resolved using a combination of Nanopore long-read and Illumina short-read sequencing. The genome consists of a single, circular chromosome of 5,004,491 bp and a plasmid of 40,722 bp.

## ANNOUNCEMENT

*Rouxiella badensis* is a Gram-negative bacterium in the family *Yersiniaceae* (1) found associated with soil and plant ecosystems (2), with emerging applications including post-harvest preservation of berries (3), a human antidepressant probiotic (4 – 6), biosynthesis of L-asparaginase (7), and uncharacterized antibiotics (8). High-quality genome sequences, such as described here, allow improved identification of metabolic pathways and secondary metabolite prediction (9, 10).

This study resolves the genome of *R. badensis* DAR84756, isolated in 2011 from a soil sample from Orange, NSW, Australia (DD latitude −33.2452, longitude 149.11586). Isolation was by addition of equal volume sterile ultrapure water to soil, which was diluted 1:10, plated onto potato dextrose agar (PDA), and incubated at 25°C for 24 hours. Single colonies were isolated by streak plate onto PDA, from which reference −80°C freezer stocks were produced and stored in 50% glycerol/PD broth.

DNA for Illumina and Nanopore libraries was prepared by the same method, from separate genomic DNA extractions, each from a single colony inoculated into 2 mL of PD broth, incubated overnight at 28°C in a shaking incubator at 200 rpm. Genomic DNA preparations were made from this with the Wizard genomic DNA purification kit (Promega, Australia) following the manufacturer’s instructions for Gram-negative bacteria. DNA quality and quantity were assessed by UV spectrometry and agarose gel electrophoresis. Frozen DNA was sent for library preparation and sequencing.

The Nanopore library was generated using the SQK-LSK109 kit (Oxford Nanopore, UK) without DNA shearing and sequenced on the GridION platform using a FLO-MIN106 (R9.4.1) flow cell at the Garvan Institute of Medical Research, Australia. Reads were called and quality controlled by MinKNOW version 20.06.17 and Guppy version 4.0.11 (11) with default settings. The Illumina DNA library was generated by fractionation and size selection on Pippin Prep (Sage Science, USA) with sequence generated on the MiSeq platform producing 2,885,611 paired reads, each 251 bp in length.

Genome assembly was completed in two sequential steps. The first was *de novo* assembly of the genome using Flye assembler (12) version 1.2 with Nanopore long DNA sequences, running on Geneious Prime 2022.1.1 software platform with default settings, except polishing was set to five iterations. Flye assembler resolved two circular contigs: a chromosome of 5,002,930 bp and a plasmid of 40,696 bp.

In the second step, Illumina 251-bp shotgun DNA reads were aligned against the Flye assembly using the “Map to reference” option in Geneious (13) with default parameters, allowing correction of Nanopore sequencing misreads, calls, and indels (14) by leveraging the accuracy of the Illumina short reads, resulting in 1,610 changes across the entire genome. The final, polished genome consisted of a circular chromosome of 5,004,491 bp and a plasmid of 40,722 bp (see Table 1).

**TABLE 1:** Summary of DNA sequence assembly data

Description	*R. badensis* DAR84756	Plasmid Sp1
GenBank accession no.	CP114062.1	CP114061.2
Chromosome size (bp)	5,004,491	40,722
GC content (%)	52.80%	49.30%
ONT sequencing		
No. of reads	268,783	
Read length (mean) (bp)	7,868	
Read *N*_50_	19,429	
Illumina sequencing		
No. of reads	2,885,611	
Read length (bp)	2 × 251	
Genome statistics		
Mean coverage (x)	414	
tRNAs	78	
rRNA (5S, 16S, 23S)	8, 7, 7	
Pseudo genes	39	
Genes (RNA)	110	
Coding genes	4,466	
Total genes	4,615	

*R. badensis* DAR84756 whole genome average nucleotide identity was 99.63% compared to *R. badensis* DSM100043^T^ (accession number CP114060 .1) confirming it to be the same species (15, 16). However, plasmid sizes differed significantly being 40,722 bp and 116,582 bp, respectively.

The NCBI Prokaryotic Genome Annotation Pipeline (17) v6.4 annotation predictions and summary are shown in Table 1.

## Data Availability

The whole genome sequence of *R. badensis* DAR84756 has been deposited in GenBank under the accession no. CP114062.1 and for its plasmid Sp1 CP114061.2 (18) with the BioSample accession number of SAMN32064793. The associated BioProject number for this project is PRJNA909035. The raw Illumina data for this genome are available from NCBI with accession number SRX19145821. The raw Oxford Nanopore data are available through accession number SRX19145820.
